# Prevalence and density of *Elaphostrongylus rangiferi* larvae in faecal samples of semi-domestic reindeer (*Rangifer tarandus tarandus*) in Norway 2013-16

**DOI:** 10.1186/s13028-025-00793-x

**Published:** 2025-01-21

**Authors:** Terje Domaas Josefsen, Torill Mørk, Ingebjørg Helena Nymo, Javier Sanchez Romano, Morten Tryland

**Affiliations:** 1 Faculty of Bioscience and Aquaculture, Nord University, Bodø, N-8026 Norway; 2 Section of Food Safety and Animal Health Research, Norwegian Veterinary Institute, Tromsø, N-9016 Norway; 3 Department of Arctic and Marine Biology, UiT The Arctic University of Norway, Framstredet 39, Breivika, Tromsø, N-9019 Norway; 4 Department of Forestry and Wildlife Management, University of Inland Norway, Koppang, N-2480 Norway

**Keywords:** Antiparasitic treatment, Baermann, Brainworm, Epidemiology, Ivermectin, LPG

## Abstract

**Background:**

The reindeer brainworm, *Elaphostrongylus rangiferi*, is a protostrongylid parasite of reindeer that has caused severe disease outbreaks in reindeer husbandry. *E. rangiferi* is considered ubiquitous in Norway, though most published prevalence studies are from Finnmark county only. In the present study, faecal samples were collected over three winter seasons (2013–2016) from eight herds of semi-domesticated Eurasian tundra reindeer (*Rangifer tarandus tarandus*) distributed all over the reindeer herding area in Norway. Baermann’s technique was used to identify *E. rangiferi* larvae. The density of larvae was measured by counting and calculating number of larvae per gram faeces (LPG) in positive samples.

**Results:**

*E. rangiferi* larvae were found in 109 of 355 faecal samples (31%). All herds had positive samples at all sampling sessions. Prevalence in adults (> 1.5 years, *n* = 176) was 43% with significant difference between herds, varying from 25 to 78%. Prevalence in calves (< 1 year, *n* = 179) was 18%, and varied with sample month, being 10% in Oct-Jan (*n* = 153) and 69% in Mar-May (*n* = 26). Prevalence did not show statistically significant difference between males and females. LPG showed a highly skewed distribution, total median LPG being 38, range 2-700. Calves in Mar-May had significantly (*P* = 0.01) higher number of LPG (median 104) than calves in Oct-Jan (median 14) and adults (median 32). LPG did not differ significantly between males and females nor between herds, but there was a significant moderate positive correlation between herd prevalence and LPG (Spearman rho = 0.35, *P* < 0.01). Samples from individually marked adult reindeer with known age (*n* = 81) showed no significant difference in prevalence between young (2–5 years) and old (6–15 years) reindeer. LPG tended to be slightly reduced with age, but the reduction was not statistically significant (*P* = 0.07). Systematic yearly treatment with ivermectin was done in four herds, but any effect on *E. rangiferi* prevalence was not clear.

**Conclusions:**

This study confirms that *E. rangiferi* was ubiquitously present in all parts of the reindeer herding area in Norway during the study period. Differences in prevalence between herds were significant, and LPG in herds increased with increasing herd prevalence.

## Background

The reindeer brainworm, *Elaphostrongylus rangiferi*, is a protostrongylid nematode with reindeer (*Rangifer tarandus* sspp.) as definitive host. The nematode was first described in Kazakhstan in the former USSR [1]. The nickname “brainworm” is due to its migration to the brain and spinal cord during development. In heavy infections clinical signs from the central nervous system (CNS) may develop, most often seen as ataxia and paresis in hind legs or all four legs [2–4]. In areas were reindeer roam on pastures used by sheep and goats, these species may also be infected and develop similar clinical CNS signs [5, 6]. Goats seem to be more vulnerable to *E. rangiferi* infection than sheep [7, 8]. Climate change may increase the occurrence of clinical outbreaks [9]. A comprehensive review of infection with *E. rangiferi* in reindeer was published in 2020 [10].

The life cycle of *E. rangiferi* is indirect, with gastropods (snails or slugs) as intermediate hosts. Reindeer pass first stage (L1) larvae in faeces, and the larvae infect the intermediate host by penetrating into the gastropod body [11]. The L1 larvae then go through a temperature dependent development to infective third stage (L3) larvae in the intermediate host. A new reindeer is infected by incidental ingestion of snails with L3 larvae during grazing. Once inside a new host, the L3 larvae penetrate venules in the abomasal wall and travel in the venous bloodstream, first to the liver, then to the lungs and then through the general circulation to all parts of the body [12]. Experimental evidence suggests that only those larvae able to reach the brain and spinal cord will survive and continue their development to L5 and mature worms [12]. The adult worms leave the CNS along the spinal nerve roots, and settle in fasciae and intermuscular connective tissue where they start to reproduce [12]. Eggs are deposited in veins and carried by the blood to the lungs where they hatch and develop into L1 larvae. The larvae penetrate into alveolar lumen and move with mucus up bronchi and trachea to the pharynx, where they are swallowed and passed in faeces. The prepatent period in an experimental infection was 4-4.5 months [4].

At present, the only feasible way to diagnose *E. rangiferi* infection in live animals is to examine faeces for the presence of L1 larvae. Thus, prevalence studies have been based on examination of faecal samples by variants of Baermann’s technique, and *Elaphostrongylus* larvae have been identified by their characteristic morphology with a kinked tail and a dorsal spine. Other protostrongylids, like *Muellerius capillaris* and *Varestrongylus* spp., have similar morphology of L1, and measurements of length and width are necessary to differentiate *Elaphostrongylus* from these species [10, 13]. However, such measurements have not been considered necessary in reindeer as no other protostrongylid than *Elaphostrongylus* is known to occur in reindeer in Europe [14].

*E. rangiferi* is considered ubiquitous in reindeer in Norway, though most prevalence studies have been done only in Finnmark county in the northernmost part of Norway.

In one study, faecal samples were collected in April for four consecutive years (1975-78) in the same herd in Finnmark. Prevalence ranged from 7 to 68% in calves and 33 to 100% in older animals [15]. In another study, faecal samples from 1470 reindeer from six different ranges along the Porsanger fjord in Finnmark were collected in the years 1977-78. The prevalence of *E. rangiferi* L1 larvae varied from 30 to 90% in reindeer 2 years or older [16].

In a more recent study, faecal samples (*n* = 114; 78 calves, 36 adults) from two different herds in Finnmark, taken in October and January, showed a total prevalence of protostrongylid infection of 40% (46/114) [17]. However, the variation between the two herds was huge; the herd sampled in October had only one positive sample (1/46, 2%), while the herd sampled in January had 45 positive samples (45/68, 66%).

The only prevalence study in semi-domestic reindeer outside Finnmark was done in Trøndelag county, in the southern part of the reindeer herding area [18]. Faecal samples from 319 semi-domestic reindeer were examined, and a prevalence from 75 to 81% were found in age classes of reindeer from 1 year to > 2 years [18]. The prevalence in calves sampled in winter were considerably lower (24%).

Another prevalence study from southern Norway was carried out in wild reindeer [14]. Results showed a prevalence in yearlings and adults of 46% (76/176). Calves were not included in this prevalence as the sampling was done in August-September, where the majority of infected calves are expected to be in the prepatent period, not yet started to shed larvae.

*E. rangiferi* is well known also in Sweden [2] and Finland [19], but no larger prevalence studies have been performed in these countries.

Antiparasitic treatment in reindeer husbandry is primarily aimed at warble fly (*Hypoderma tarandi*) and throat bot (*Cephenemyia trompe*) larvae. The drug of choice is ivermectin, which is known to be highly effective against warble and throat bot larvae [20, 21], and the treatment is done in late autumn or winter when reinfection of these parasites no longer may occur. The effect of ivermectin treatment on *E. rangiferi* is considered moderate or low [20, 21]. Ivermectin does not normally penetrate into the CNS [22]. Thus, the timing of treatment is not optimal to target new infections of *E. rangiferi*, as the majority of the new larvae are expected to be developing in the brain and spinal cord during late autumn and mid-winter. Despite this theoretical basis, calves treated with ivermectin on 25 November showed an *E. rangiferi* prevalence of 0% (0/5) in treated calves, compared to 80% (4/5) in untreated calves [23].

**Fig. 1:**
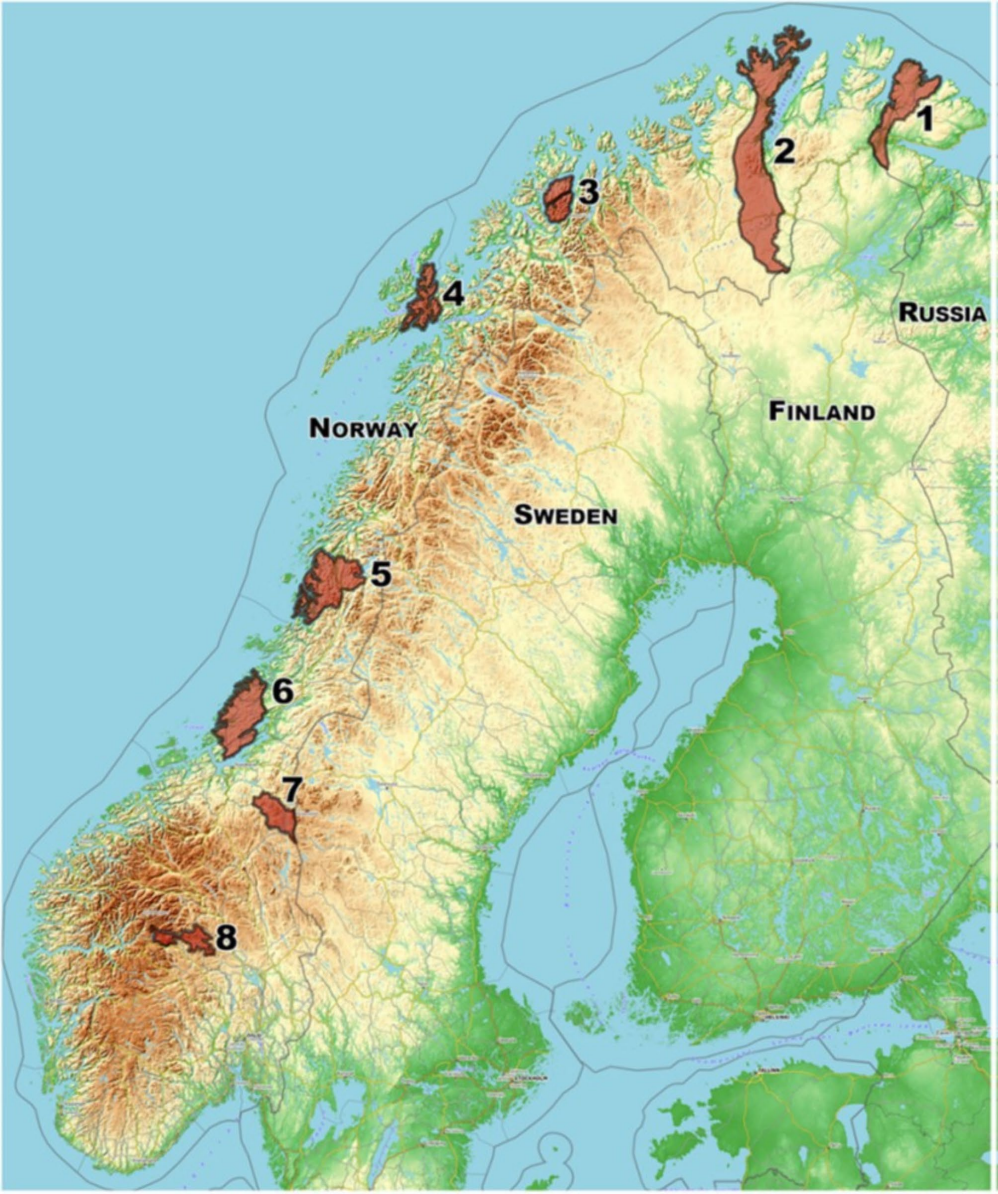
Map showing the location of the eight reindeer herds (Herd 1–8) sampled in this study. (Figure from [24]). Name of geographical areas: Herd 1: Deanu/Tana, Finnmark county. Herd 2: Lakselv, Finnmark county. Herd 3: Tromsø, Troms county. Herd 4: Lødingen, Nordland county. Herd 5: Hattfjelldal, Nordland county. Herd 6: Fosen, Trøndelag county. Herd 7: Røros, Trøndelag county. Herd 8: Filefjell, Innlandet county

The present study was part of a larger study where eight semi-domestic reindeer herds in different parts of Norway (Fig. 1) were sampled for different issues several times through the years 2013–2016. The aim of the present study was to investigate the prevalence of *E. rangiferi* infection in the whole reindeer herding area in Norway, and the density of larvae in faeces of infected animals.

## Methods

### Herds and sampling

Faecal samples were collected from eight herds (Herd 1–8) of semi-domesticated Eurasian tundra reindeer (*Rangifer tarandus tarandus*), located in different parts of the reindeer herding area in Norway (Fig. 1). The herds were numbered in order from north (Herd 1 and 2) to south (Herd 8). Samples were collected from each herd during three consecutive winter seasons (2013-14, 2014-15 and 2015-16). The samples were collected from rectum of physically restrained live reindeer or from rectum of slaughtered reindeer post mortem (Herd 4 only). Due to practical limitations, faecal samples were not collected from all herds each year. In some animals none or very little faeces could be obtained. Samples smaller than 1 g were excluded from the study.

A total of 355 faecal samples were collected, with 115, 118 and 122 samples in each of the three winter seasons. Sex and age class (< 1 year = calf, > 1.5 year = adult) were registered at sampling. Some animals were individually marked, enabling exact age of adults. Faecal samples were most often collected in winter (October-March), but at one occasion in spring (early May; Herd 3). The distribution of samples on herds, age class and sampling month is shown in Table 1.

**Table 1:** Number of reindeer faecal samples analysed, by herd, sampling month and age class

Herd	Oct		Nov		Dec		Jan		Mar		May		Sum		Total
	C	A	C	A	C	A	C	A	C	A	C	A	C	A	
1					6	43							6	43	49
2					17	36							17	36	53
3			13	7	10	2					21		44	9	53
4	12	7			10	7							22	14	36
5			15	2	29	15							44	17	61
6							9	10	5	11			14	21	35
7			10	7			4	13					14	20	34
8					18	16							18	16	34
Sum	12	7	38	16	90	119	13	23	5	11	21	0	179	176	355

C = Calf (< 1year). A = Adult (> 1.5 years). Samples were collected in 2013–2016. February and April are omitted, as no animals was sampled these months

The faecal samples were frozen at -20 °C within 2 days after sampling and kept frozen up to three years before analysed.

Routines for antiparasitic treatment were registered by interview of the owners in retrospect.

### Analysis of faecal samples

A modified Baermann method [25] was used for faecal analysis. Samples of 5 g faeces were wrapped in a single layer of gauze and hung up by use of a stick in a disposable plastic wine glass with a funnel-shaped bottom. The glass was filled up with lukewarm tap water covering the sample, and kept at room temperature 18–24 h. The faecal sample was then removed, and excess water removed by a sucking pump. The bottom 3–5 mL of water and sediment was transferred to a centrifuge tube and centrifuged (600x g, 3 min). Supernatant was removed, leaving 2 mL of water and sediment in the tube. The bottom sediment was then thoroughly mixed with the fluid, and a subsample of 200 µL was transferred onto an object glass. The subsample was examined in a microscope using 10x objective, and larvae with typical morphology for *Elaphostrongylus* L1 larvae (kinked tail with dorsal spine), were counted. In positive samples, the number of larvae per gram faeces (LPG) was calculated, based on the initial weight of the sample. If the first subsample was negative, another subsample of 200 µL was examined, making the theoretical detection limit 1 LPG in a 5 g faecal sample.

About one third of the samples were smaller than 5 g (56 samples 1-2.9 g, 69 samples 3-4.9 g). When analysing these samples, the weight was noted and the procedure was continued as if the sample was 5 g, thereby accepting a higher detection limit (5 LPG in a 1 g sample). If samples were positive, LPG was calculated using the initial weight of the sample.

### Statistics

Result variables in this study were prevalence of positive samples and LPG in positive samples. Chi-squared test (or Fisher-Freeman-Halton Exact test where expected values were below five) was used to compare differences in prevalence between groups, while Mann-Whitney U test and Kruskal-Wallis test were used to compare LPG between two or more groups, respectively. Spearman rho was used to explore relationship between LPG and herd prevalence or age. Level of significance was *P* = 0.05. The tests were conducted using IBM SPSS Statistics version 29.0.2.0 (20).

## Results

Of 355 analysed faecal samples, 109 (31%) were positive for *E. rangiferi* larvae (Table 2). All herds had positive samples at all sampling sessions.

**Table 2:** Analyses of 355 reindeer faecal samples for L1 larvae of *Elaphostrongylus Rangiferi*

	*n*	*n* pos	Prevalence (%)	95% CI	Median LPG	Mean LPG	Min-Max
Calves (all)	179	33	18	13–24	44	128	2-700
Calves in Oct-Jan	153	15	10	5–15	14	74	2-421
Calves in Mar-May	26	18	69	51–87	104	173	10–700
Adults	176	76	43	36–50	32	70	3-660
Total	355	109	31	26–36	38	88	2-700

Data are stratified on age class and, for calves, also sampling season. Calves < 1year, adult > 1,5 year, n = number of faecal samples analysed, n pos = number of positive samples, CI = confidence interval, LPG = larvae per gram faeces

**Fig. 2:**
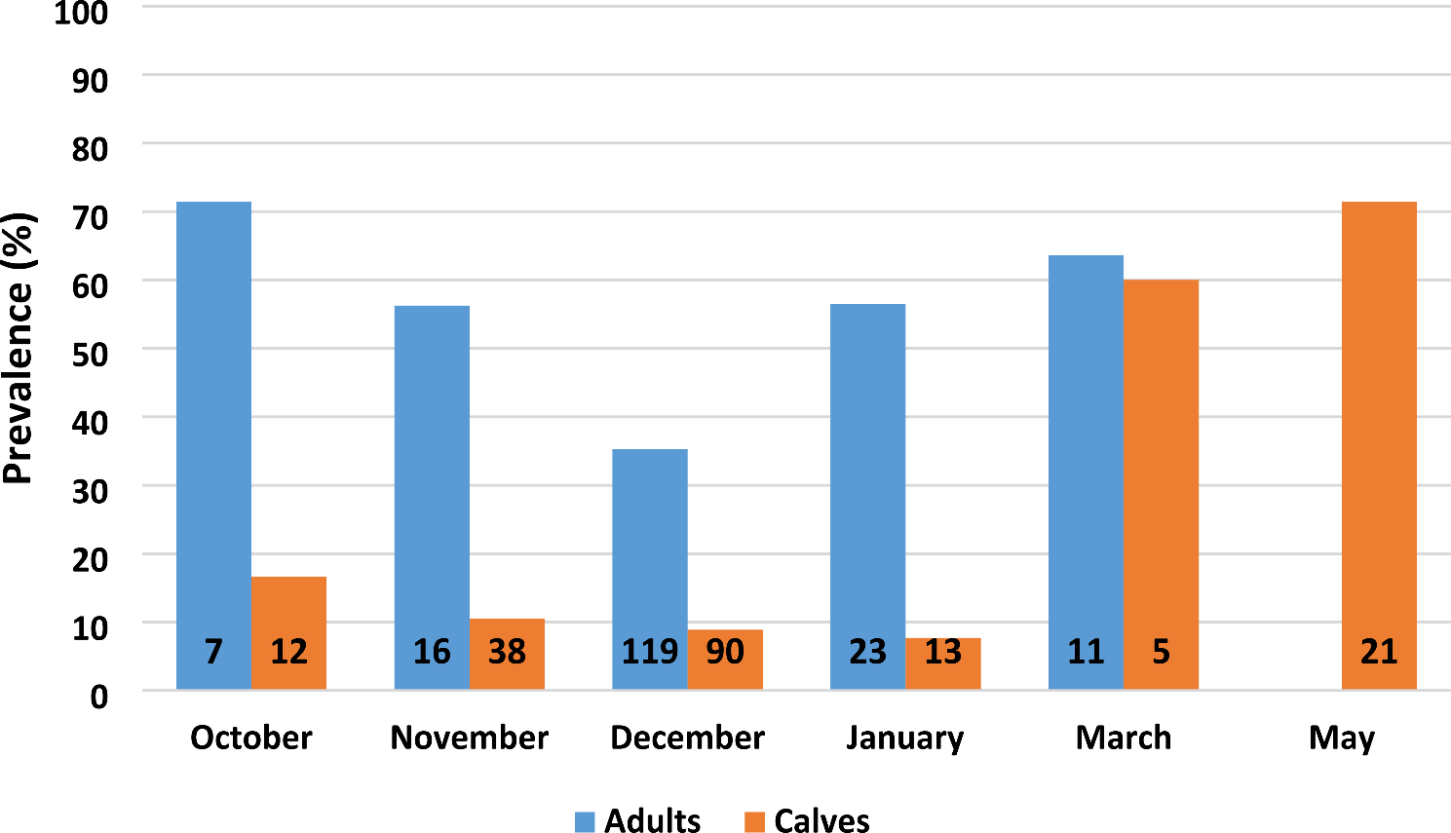
Prevalence of *E. rangiferi* larvae in reindeer faecal samples (*n* = 355) distributed on sample months. Numbers on each bar represent the number of animals examined. Calves < 1 year, *n* = 179, Adults > 1.5 years, *n* = 176. February and April are omitted as no animals were sampled these months

Samples were collected from October to May, with December being the month with most samples (68% of samples from adults, 50% of samples from calves; Table 1). Sampling month influenced prevalence in calves, being low in autumn and winter (Oct-Jan), with a significant (*P* < 0.001) increase in spring (Mar-May; Table 2; Fig. 2). Prevalence in adults was significantly (*P* = 0.002) lower in December compared to the grouped prevalence of adults in the other months (Fig. 2). The low December prevalence must be seen in connection with differences in herd prevalence (see discussion).

The distribution of LPG was skewed, with most animals having low or moderate numbers of LPG, and a few animals having high numbers of LPG. Median LPG was 38, range 2-700 (Table 2). In adult reindeer, there was no significant difference in LPG between different sampling months. Calves in March-May had significantly higher number of LPG than calves in October-January (*P* = 0.01) and adults (*P* = 0.01; Table 2).

There was no significant difference in prevalence or LPG between males and females. Prevalence in males exceeded that of females, particularly in calves (Table 3), but still not statistically significant (*P* = 0.07 for sum of calves).

**Table 3:** Prevalence of *E. Rangiferi* L1 larvae in male and female reindeer faeces

Age class	Prevalence (%) in males	95% CI	Prevalence (%) in females	95% CI
Calves in Oct-Jan	11 (8/76)	4–17	8 (5/65)	1–14
Calves in Mar-May	78 (14/18)	59–97	50 (4/8)	15–85
Sum calves^a^	23 (22/94)	15–32	12 (9/73)	5–20
Adults > 1.5 year	50 (12/24)	30–70	42 (64/152)	34–50

^a^Twelve calves omitted as sex was not registered

CI = confidence interval

**Fig. 3:**
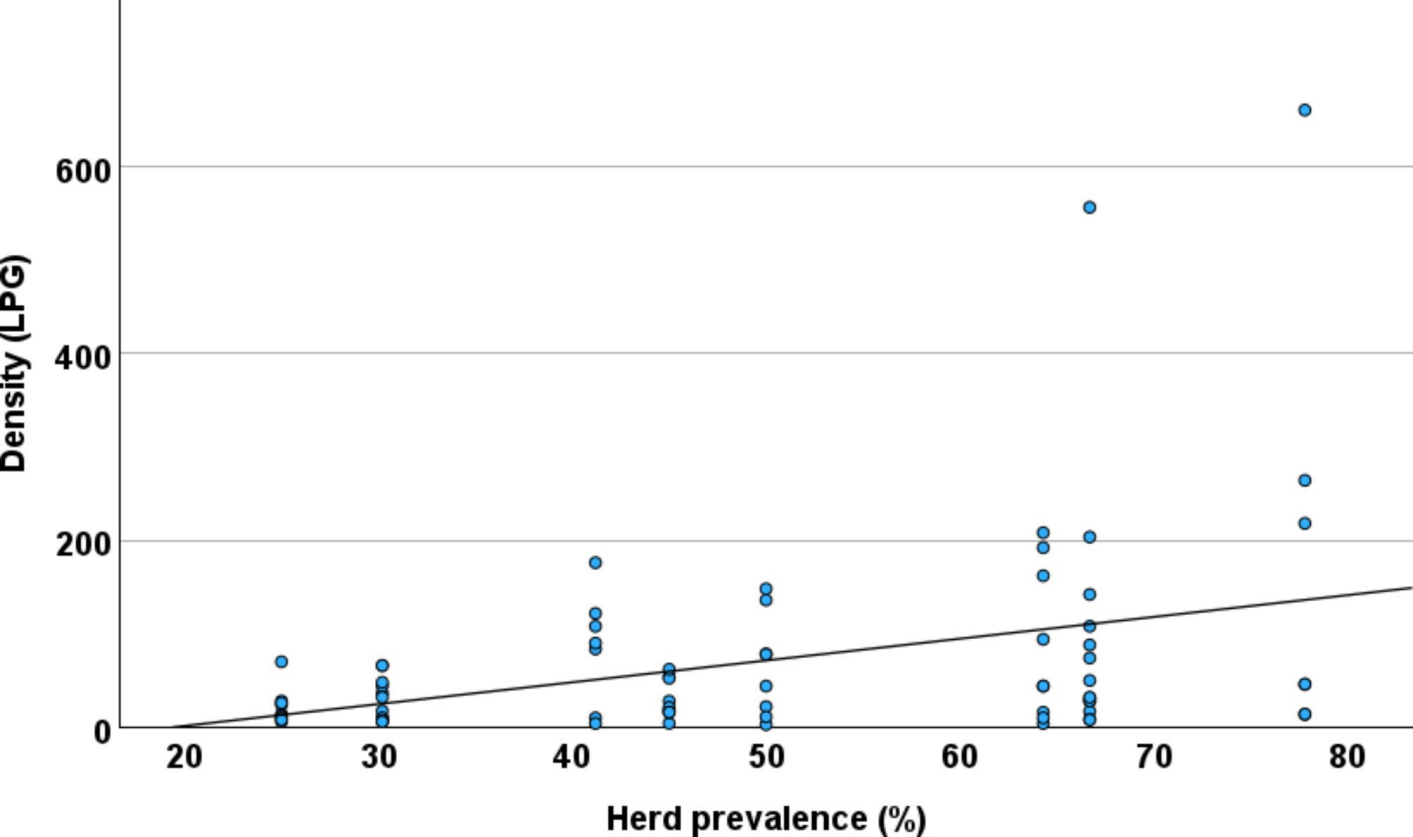
Variation in density of faecal larvae (LPG) in relation to herd prevalence. Herd prevalence (Herd 1–8) is taken from Table 4. Numbers of LPG are from all adult reindeer positive for *E. rangiferi* L1 larvae in faeces (*n* = 76). Spearman rho = 0.35, *P* < 0.01

Due to variation in proportion of calves sampled in each herd (Table 1), and the huge influence of sample month on calf prevalence (Fig. 2), calves are excluded from the comparison of herds. Prevalence in adults ranged from 25 to 78% between different herds (Table 4). Comparing all herds showed that differences in prevalence between herds were statistically significant (*P* = 0.01), while differences in LPG between herds were not (*P* = 0.09). There was a moderate positive correlation between herd prevalence and the density of the larvae in faeces (LPG) (Spearman rho = 0.35, *P* < 0.01; Fig. 3).

**Table 4:** Herd prevalence (%) and density (LPG) of *E. Rangiferi* larvae in faeces from adult reindeer

Herd	Prevalence in adults			Median LPG in adults		Antiparasitic treatment
	n	Prev. (%)	95% CI	n pos	Median LPG	
1	43	30	17–44	13	17	All animals treated at winter gathering
2	36	25	11–39	9	14	All calves treated, some in September, all in December
3	9	78	51–100	7	46	Sporadic treatment of some animals, no systematic treatment
4	14	64	39–89	9	44	Sporadic treatment of some animals, no systematic treatment
5	17	41	18–65	7	90	All animals treated at winter gathering
6	21	67	47–87	14	41	All animals treated at winter gathering
7	20	45	23–67	9	21	No treatment
8	16	50	26–75	8	61	No treatment
All herds	176	43	36–50	76	32	

Antiparasitic treatment (if done) is given in the form of subcutaneous injection of ivermectin. n = number of faecal samples analysed, n pos = number of positive samples, CI = confidence interval

Routines for antiparasitic treatment in the herds are listed in Table 4. Subcutaneous injection of ivermectin was used in all herds where treatment was done. No clear association between antiparasitic treatment and herd prevalence was evident. The three herds with lowest prevalence had implemented routine treatment of calves (Herd 2) or all reindeer (Herd 1 and 5), but so had Herd 6, with the second highest prevalence of *E. rangiferi* larvae.

Any distinct association between prevalence and latitude could not be observed (Table 4).

Of 176 faecal samples from adult reindeer, 81 were from reindeer with known age, ranging from 2 to 15 years. The prevalence of positive samples was the same in adults with and without known age (43%; 35/81 and 41/95 respectively).

Reindeer with known age were not evenly distributed between herds and age classes. To investigate if prevalence was influenced by age, the herds were divided into three levels (high, medium and low prevalence), and prevalence was calculated separately for young (2–5 years) and old (6–15 years) adult reindeer.

**Fig. 4:**
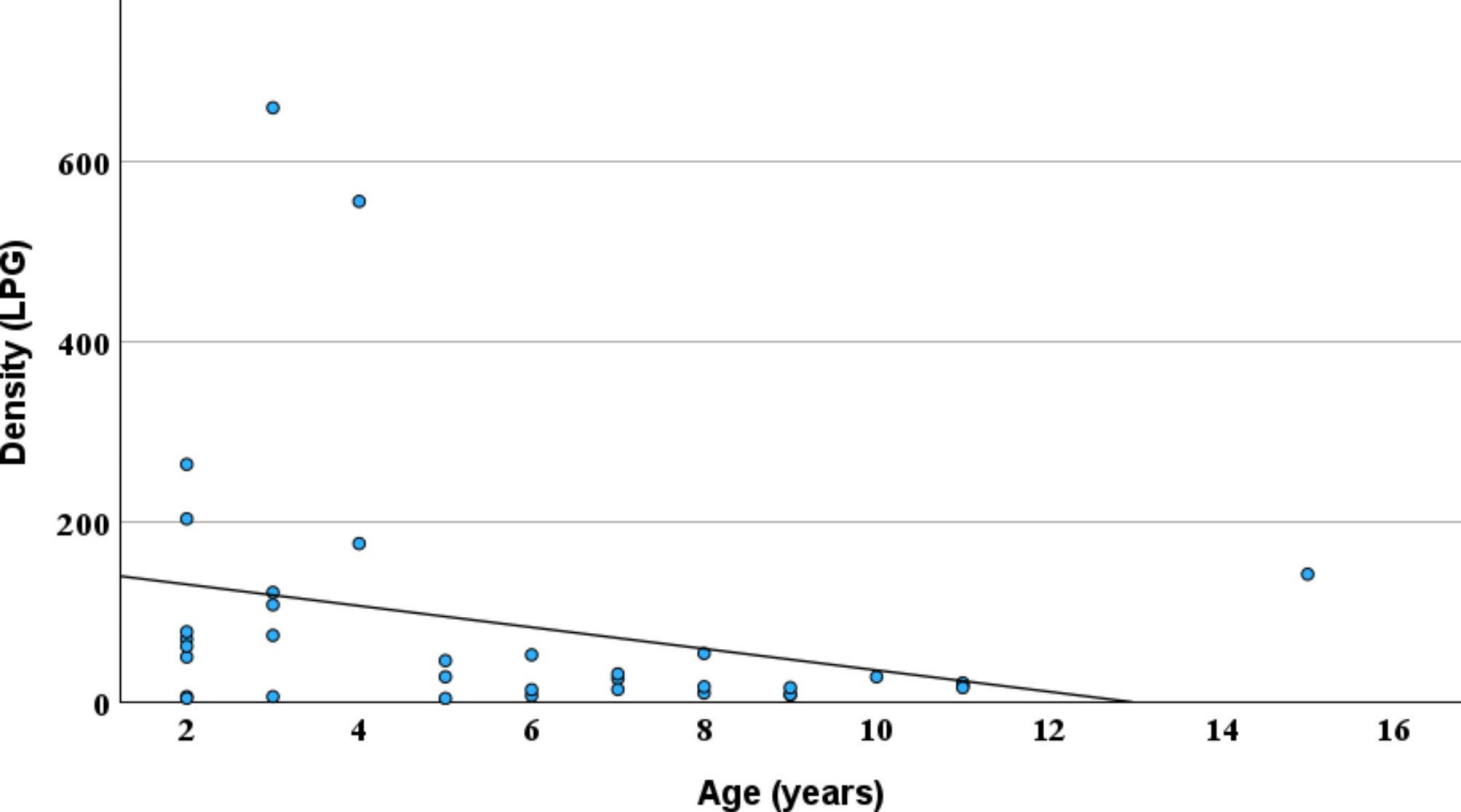
Density of *E. rangiferi* larvae (LPG) plotted against age in adult reindeer (*n* = 35). Spearman rho=-0.32, *P* = 0.07

There was no significant difference in prevalence between young (2–5 years) and old (6–15 years) adult reindeer (Table 5). There was a trend of decreasing LPG with age (Spearman rho = -0.32, Fig. 4), but the decrease was not statistically significant (*P* = 0.07).

**Table 5:** Influence of age on prevalence of *E. rangiferi* L1 larvae in faeces of 81 adult reindeer

	2–5 years			6–15 years		
Prevalence level	n	Prevalence (%)	95% CI	n	Prevalence (%)	95% CI
High (> 60%) (Herd 3, 4 and 6)	11	73	46–99	10	60	30–90
Medium (40–60%) (Herd 5, 7 and 8)	14	50	24–76	14	50	24–76
Low (< 40%) (Herd 1 and 2)	9	33	3–64	23	17	2–33
Total	34	53	36–70	47	36	22–50

Due to herd differences in prevalence and uneven distribution of reindeer with known age, the herds are grouped into three prevalence levels (high, medium and low). n = number of faecal samples analysed, CI = confidence interval

In adults the number of samples with sample weight below 5 g was fairly evenly distributed across herds, justifying merging all herds to investigate the effect of smaller samples. In calves, small samples occurred proportionately more frequent in spring, making it necessary to divide calf samples after sampling months (Table 6). In adults and calves in October-January prevalence of positive samples tended to be higher in 5 g samples than in smaller samples, but the difference was statistically significant only for calves in October-January (*P* = 0.047). For calves in March-May no trend was clear (Table 6).

**Table 6:** Influence of faecal sample weight on prevalence (%) and density (LPG) of *E. rangiferi* larvae

Sample weight	Adults					Calves in Oct-Jan					Calves in Mar-May				
	n	n pos	P (%)	95% CI	Median LPG	n	n pos	P (%)	95% CI	Median LPG	n	n pos	P (%)	95% CI	Median LPG
5 g	123	57	46	38–55	44	94	13	14	7–21	14	13	9	69	44–94	38
3–4.9 g	28	11	39	21–57	17	34	0	0		0	7	5	71	38–100	300
1–2.9 g	25	8	32	14–50	44	25	2	8	0–19	257	6	4	67	29–100	174
Total	176	76	43	36–50		153	15	10	5–15		26	18	69	51–87	

Adult > 1.5 year. Calves < 1year. n = number of faecal samples analysed, n pos = number of positive samples, P = prevalence, CI = confidence interval

In adults, LPG did not show any association with sample weights. In calves, LPG was significantly higher in small samples compared to 5 g samples (calves in Oct-Jan *P* = 0.04; calves in Mar-May *P* = 0.02; Table 6).

## Discussion

The present study confirms that *E. rangiferi* was ubiquitous in the reindeer herding area in Norway 2013-16, with an overall prevalence of 43% in reindeer 1.5 years or older. Though the samples are taken about ten years ago, we believe they still are representative, or at least not too high, as the ongoing climate change is expected to increase the prevalence of *E. rangiferi* [9].

Positive samples were found in all herds at all sampling sessions. The prevalence of *E. rangiferi* in adults is within the same order of magnitude as previous reports [14–18].

The prevalence in calves (18%) was considerably lower than in adults (43%). This is explained by the sampling months in relation to the epidemiology and life cycle of the parasite. Reindeer calves are born in May, with a peak in the middle of the month, but with variation between herds and between years [26, 27]. Accepting four months as a minimum prepatent period [4], a calf born in May, infected within a few days after birth, should start shedding larvae in faeces in September. One wild reindeer calf shedding *Elaphostrongylus* L1 larvae in September [14] shows that such early infections do occur, and our data show positive samples from October and onwards, confirming that some calves are infected early in life. However, the low prevalence in calves in autumn/early winter (10%) suggests that early infections are not the main route of the parasite.

Early infection with *E. rangiferi* depends on ingestion of infective L3 larvae that have survived winter in the gastropod intermediate host. The winter survival in northern gastropods is believed to be high [28]. However, experimental evidence suggest that gastropods infected with *E. rangiferi* larvae that have started the development to L2 and L3, have reduced winter survival compared to gastropods infected with larvae that have been arrested in the L1 state due to low temperature [29]. Furthermore, the same study indicates that L1 larvae have significantly higher winter survival rate within the gastropod host, compared to L2 or L3 larvae. These two factors probably contribute to a low rate of infection during early summer, explaining the low prevalence in calves in autumn (October to December).

Instead, winter surviving L1-infected gastropods and newly infected gastropods during the summer result in accumulation of infective L3 larvae that causes a “wave” of new infections in late summer or autumn. Summer temperature is the main factor for determining how many L1 reach the L3 infective stage during the summer, and how early this happens [28]. In our data even the sampling on 21 January (Herd 7, 2015) and 24 January (Herd 6, 2016) showed low prevalence in calves. This suggests that the main uptake of infective L3 larvae occurred in the end of September or later in these herds those years. A high prevalence of L1 in faeces may occur in January, as illustrated by the reported prevalence of 59% (26/44) in calves in mid-January 2018 [17]. In another example, two naturally infected male reindeer calves were taken into a research facility in the autumn, and sampled every second week until they started shedding L1 larvae in March [30]. This illustrates the variation in timing of the autumn infection “wave”. In our data, the “wave” of new infections in calves was apparent in samples from March and May (Fig. 2).

The overall prevalence in adults shows variation between months, with a significantly lower prevalence in December compared to other months. A reduced prevalence in December is not supported by a previous study [30], and this finding is most probably the result of differences in prevalence between herds. The two herds with lowest prevalence (Herd 1 and 2, Table 4) both had a large number of adults being sampled in December (Table 1), while the herds with highest prevalence (Herd 3, 4 and 6, Table 4) were partly or fully sampled in other months and with a much lower number of adults (Table 1).

In our study, there was no statistically significant difference in prevalence between males and females. Still, males had slightly higher prevalence than females, particularly among calves. A slight majority of *E. rangiferi* infected males has also been previously reported in reindeer [17, 31], as well as for *Elaphostrongylus alces* in all age classes of moose (*Alces alces*) [32] or in young moose [33], but not for *Elaphostrongylus cervi* in red deer (*Cervus elaphus*) [34]. In clinical outbreaks of cerebrospinal elaphostrongylosis in reindeer, a majority of affected males have been reported [2, 35]. Thus, it may seem as if male calves are more frequently infected and/or more susceptible to the infection than female calves.

Male calves are on average heavier than female calves [36]. One study found that infected calves were heavier than uninfected [31], suggesting that the heavier calves were more dominant and gained more frequent access to otherwise scarce patches of nutritious forbs, where also gastropod density and thus, the infection risk, was presumed to be highest. The weight influence was not supported by a later study [18] who found the weight of infected calves to be equal in males and significantly lower in females compared to uninfected calves.

The density of faecal larvae (LPG) in our study, is comparable to values reported previously [15, 18, 30]. Shedding of L1 is reported to be low in summer [18, 30], and this may explain the much lower number of LPG reported in wild reindeer sampled in August/September [14].

Herd differences in prevalence were prominent in our data, ranging from 25 to 78%. Many factors may influence herd prevalence, including summer temperature and precipitation, range altitude, range use, gastropod habitats and antiparasitic treatment. The effect of summer temperature is best documented. Clinical outbreaks of cerebrospinal elaphostrongylosis are shown to be significantly associated with higher summer temperatures, while association with increased summer rainfall were less strong [37]. A reindeer herd sampled in the years 1975-78 showed decreasing prevalence, and the decrease was tributed to lower summer temperatures these years, compared to the years before [15]. The altitude of summer ranges is shown to influence *E. rangiferi* prevalence in wild reindeer [14]. This may be a direct result of lower temperatures at higher altitude, but may also be influenced by the amount of suitable gastropod habitats within the herds range [16, 18].

Routines of antiparasitic treatment did not unequivocally influence herd prevalence (Table 4). Still the three herds with lowest prevalence all performed systematic antiparasitic treatment. Even though the effect of ivermectin on *E. rangiferi* is characterized as moderate, results have shown a significant reduction in both prevalence and LPG [20]. Red deer treated with anthelmintics had significantly lower prevalence of *E. cervi* (39%) than untreated (74%), but no details of the anthelmintic treatment is provided [34].

Differences in LPG between herds were not statistically significant, but there was a significant trend towards higher number of LPG in herds with higher prevalence (Fig. 3). This finding seems logical, as high infection pressure leading to high prevalence could also be expected to lead to a generally higher intensity of infection in the herd, resulting in higher density of faecal larvae.

An interesting finding in this study is that both prevalence and density (LPG) seem to stabilize reindeer older than 5 years (Table 5; Fig. 4). Previous studies have shown that prevalence of *E. rangiferi* infection and LPG reach a plateau at 3–6 years of age, prevalence being 90–100% [38, 39]. In red deer, prevalence of *E. cervi* reaches a peak at about 6 years of age, and is maintained at 70–100% once this age is reached [34]. Thus, in both these studies prevalence reached relatively high levels before stabilizing. Our results seem to indicate that prevalence may stabilize at lower herd prevalence levels (Table 5). *E. rangiferi* is considered a long-lived nematode [30], and particularly in the herds with low prevalence rates (Herd 1 and 2), the prevalence could be expected to increase with age as more reindeer become infected. Contrary to this expectation, the prevalence in these herds tended to be reduced with age. The reason for this is unclear, but again, a possible effect of systematic antiparasitic treatment with ivermectin cannot be ruled out as a contributing cause.

A slight decrease in LPG with age in our study (Fig. 4) is in accordance with previous findings in reindeer [30], and in female, but not male, red deer [34]. The decrease may be related to mortality and/or reduced fecundity of elderly nematodes, as shown in *Parelaphostrongylus tenuis* in white-tailed deer (*Odocoileus virginianus*) [40, 41].

In the Baermann method, samples of 10 g are commonly recommended [25], but samples down to 5 g are frequently used for reindeer [17, 18]. In our study, 90% of the faecal samples were collected from rectum of physically restrained live reindeer, and sufficient sample material was sometimes difficult to achieve. For this reason, we decided to use samples down to 1 g. The results in Table 5 indicate that reduced sample weight may have led to lowered prevalence, presumably due to increased detection limit. In calves in spring, there is no change in prevalence associated with sample weight, possibly because the number of LPG is higher at this time of year (Table 2), so that a slight reduction in detection limit does not matter.

There was no significant association between LPG and sample size in adults, but in calves there was a significant increase in LPG when samples were smaller (Table 5). We have no satisfactory explanation for this finding. Small sample weight may be caused by nearly empty rectum due to recent defecation or by generally lowered faecal production. If small samples are associated with lowered faecal production, an increased density of larvae could be expected, as seen in the calves. The lack of association between small samples and LPG in adults could then indicate that the small samples in adults occur more incidentally, due to recent defecation.

## Conclusions

This study confirms that *E. rangiferi* was present in all parts of the reindeer herding area in Norway in the study period. Prevalence in adults varied from 25 to 78% between herds. LPG in herds increased with increasing herd prevalence. Prevalence stabilized and density of faecal larvae (LPG) tended to decrease in reindeer older than five years.

## Data Availability

The dataset used during the current study are available from the corresponding author on reasonable request.

## Declarations

### Prior publication

Data have not been published previously.
